# Complement Factor B Mediates Ocular Angiogenesis through Regulating the VEGF Signaling Pathway

**DOI:** 10.3390/ijms22179580

**Published:** 2021-09-03

**Authors:** Hannah Murray, Beiying Qiu, Sze Yuan Ho, Xiaomeng Wang

**Affiliations:** 1 Institute of Molecular and Cell Biology, Agency for Science Technology and Research (A*STAR), Proteos, 61 Biopolis Dr., Singapore 138673, Singapore; murrayh@imcb.a-star.edu.sg; 2 Duke-NUS Graduate Medical School, 8 College Road, Singapore 169857, Singapore; beiying.qiu@duke-nus.edu.sg (B.Q.); hoszeyuan@duke-nus.edu.sg (S.Y.H.); 3 Singapore Eye Research Institute, The Academia, 20 College Road, Discovery Tower Level 6, Singapore 169856, Singapore; 4 Lee Kong Chian School of Medicine, Nanyang Technological University Singapore, 59 Nanyang Drive, Singapore 636921, Singapore

**Keywords:** complement factor B, angiogenesis, VEGF, eye

## Abstract

Complement factor B (CFB), a 95-kDa protein, is a crucial catalytic element of the alternative pathway (AP) of complement. After binding of CFB to C3b, activation of the AP depends on the proteolytic cleavage of CFB by factor D to generate the C3 convertase (C3bBb). The C3 convertase contains the catalytic subunit of CFB (Bb), the enzymatic site for the cleavage of a new molecule of C3 into C3b. In addition to its role in activating the AP, CFB has been implicated in pathological ocular neovascularization, a common feature of several blinding eye diseases, however, with somewhat conflicting results. The focus of this study was to investigate the direct impact of CFB on ocular neovascularization in a tightly controlled environment. Using mouse models of laser-induced choroidal neovascularization (CNV) and oxygen-induced retinopathy (OIR), our study demonstrated an increase in CFB expression during pathological angiogenesis. Results from several in vitro and ex vivo functionality assays indicated a promoting effect of CFB in angiogenesis. Mechanistically, CFB exerts this pro-angiogenic effect by mediating the vascular endothelial growth factor (VEGF) signaling pathway. In summary, we demonstrate compelling evidence for the role of CFB in driving ocular angiogenesis in a VEGF-dependent manner. This work provides a framework for a more in-depth exploration of CFB-mediated effects in ocular angiogenesis in the future.

## 1. Introduction

Angiogenesis refers to the growth of new blood vessels from pre-existing vasculature [1]. During this process, endothelial cells (EC) will first branch out from a parent vessel by proliferation and migration, extending into the surrounding matrix [2]. Perivascular cells, such as smooth muscle cells and pericytes, will subsequently be recruited to the nascent vessels, which secrete extracellular matrix (ECM) proteins and provide further support to newly formed blood vessels [3,4,5]. The formation of ordered vasculature with hierarchical branching patterns requires a fine balance of molecular mechanisms [6]. A disturbance in this tightly controlled process leads to dysregulated angiogenesis and pathology ensues. Pathological angiogenesis in the eye is the leading cause of blindness worldwide, including diabetic retinopathy (DR), neovascular age-related macular degeneration (nAMD), and retinopathy of prematurity (ROP) [7]. Vascular endothelial growth factor (VEGF) is one of the most potent angiogenic mediators and has a well-established role in driving ocular angiogenesis [8]. Over the last two decades, pharmacological agents targeting VEGF have transformed the treatment landscape for ocular angiogenic diseases. However, despite clear efficacy, a substantial number of patients are intrinsically refractory to anti-VEGF therapy or may develop resistance over time [9]. Considering the important neuroprotective effect of VEGF [10] and its role in physiological angiogenesis [11], chronic VEGF suppression also raises concerns regarding potential adverse local and systemic side effects [12,13,14]. Effective management of ocular angiogenic diseases, therefore, remains a significant unmet medical need. The development of new therapies capable of preventing or slowing the onset and progression of such diseases remains a priority and this underpins the need for continuing efforts to fully elucidate the mechanisms involved in pathological ocular angiogenesis.

Traditionally, the complement system is primarily viewed as the first line of defense against microbial intruders, quickly tagging and eliminating them and buying the adaptive immune response time to pick up momentum [15]. However, there is now a new perception of complement that reaches far beyond the elimination of pathogens [16]. Numerous studies have elaborated on the pathogenic role of complement during immune, inflammatory, neurodegenerative, ischemic, and age-related diseases [17], and it is now widely accepted that the presence and activation of complement plays a crucial role in the pathogenesis of a large number of diseases, with emerging evidence that poorly controlled complement activation, particularly the alternative pathway (AP), is associated with vascular pathologies in the eye [18]. Complement factor B (CFB), a 95-kDa protein, is a crucial catalytic element of the alternative pathway (AP) of complement. After binding of CFB to C3b, activation of the AP depends on the proteolytic cleavage of CFB by factor D to generate the C3 convertase (C3bBb) [19]. The C3 convertase contains the catalytic subunit of CFB (Bb), the enzymatic site for the cleavage of a new molecule of C3 into C3b. In addition to its role in activating the alternative pathway there is now increasing evidence implicating the involvement of CFB in ocular angiogenic diseases. CFB polymorphisms have been associated with nAMD [20,21,22,23,24,25,26]. However, the exact functional significance of this association and the cell type that CFB acts on remain unclear. The mechanisms involved in the crosstalk between CFB and neovascularization are also ambiguous. For instance, a pro-angiogenic function of CFB has been reported in the mouse model of laser-induced CNV, which represents a model of angiogenesis in nAMD [24,25,26,27,28,29]. In contrast, Cfb-/- mice demonstrated significantly more retinal neovascularization following OIR treatment, which suggests that CFB has a protective role in DR by aiding in the clearance of pathological neo-vessels [30]. Since multiple cells present in the ocular microenvironment contribute to abnormal blood vessel formation in vivo, it is challenging to dissect the cell-specific role of CFB in complex animal models. The overarching aim of this study was to establish the direct impact of CFB on ocular angiogenesis in a tightly controlled environment using various in vitro and ex vivo preclinical models.

## 2. Results

### 2.1. Cfb Expression Is Up-Regulated in Mouse Models of Neovascular Ocular Disease

**Figure 1:**
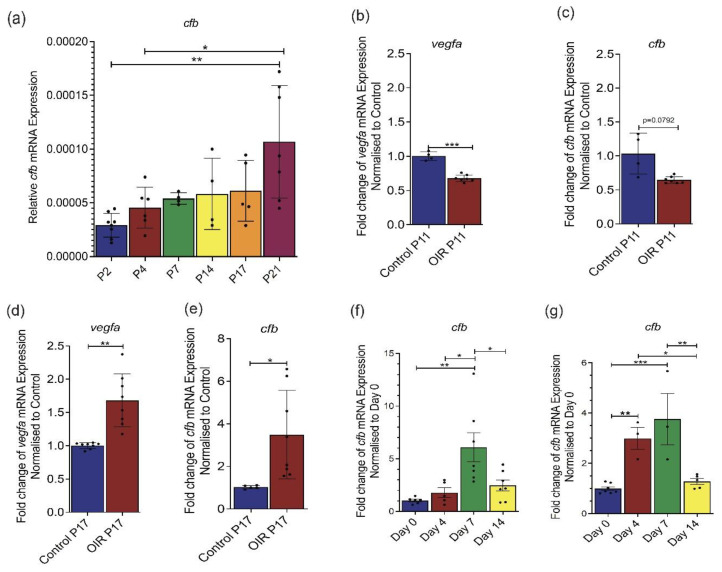
*Cfb* expression is upregulated in ocular tissues of mouse models of ocular angiogenesis. Relative gene expression of (**a**) *Cfb* in the developing retina of C57BL/6 mice (n = 8 P2, n = 6 P4, n = 5 P7, n = 4 P14, n = 5 P17, n = 7 P21); relative gene expression of (**b**) *Cfb* and (**c**) *Vegf* in P11 (hyperoxic) retina of C57BL/6 mice subjected to oxygen-induced retinopathy (OIR) (n = 7) as compared to those in age-matched normoxia retina (n = 4); relative gene expression of (**d**) *Cfb* in P17 (angiogenic) retina of C57BL/6 mice subjected OIR (n = 7) as compared to those in age-matched normoxia retina (n = 5); relative gene expression of (**e**) *Vegf* in P17 (angiogenic) retina of C57BL/6 mice subjected to OIR (n = 8) as compared to those in age-matched normoxia retina (n = 8); relative gene expression of *Cfb* in the (**f**) retina of C57BL/6 mice subjected to laser-induced choroidal neovascularization (CNV) (n = 6 D0, n = 5 D4, n = 6 D7, n = 7 D14); relative gene expression of *Cfb* in the (**g**) choroid/RPE complex of C57BL/6 mice subjected to laser-induced CNV (n = 7 D0, n = 3 D4, n = 3 D7, n = 5 D14) as determined by RT-qPCR analysis. Results represent the mean ± standard error mean (SEM). Statistical significance was determined by unpaired, two-tailed Student’s *t*-test or one-way ANOVA followed by Tukey multiple comparisons test; *: *p* < 0.05, **: *p* < 0.01, ***: *p* < 0.001.

In contrast to humans, the mouse retina is avascular at birth, and the formation of the retinal vasculature occurs predominantly through angiogenesis in a tightly regulated process [31,32]. To establish the association of *Cfb* expression and retinal angiogenesis, retinae were harvested postnatally (P) at P2, P4, P7, P14, P17, and P21 and subjected to RT-qPCR analysis of *Cfb* expression. Our study showed a steady increase in *Cfb* expression as the retinal vasculature develops from P2 to P21 (Figure 1a). The mouse model of oxygen-induced retinopathy (OIR) mimics the clinical features of DR [33,34]. In this model, P7 pups and a nursing mum were kept in a hyperoxic chamber containing 75% O_2_ for 5 days, which not only prevents further retinal angiogenesis but also causes the regression of already formed retinal vasculature. Upon returning to room air, the avascular retinal then becomes hypoxic and triggers both normal and abnormal angiogenesis which reaches the highest level at P17. In this study, we showed that *Cfb* expression was significantly attenuated in the hyperoxic retina at P11 as compared to that in the normoxic retina (Figure 1b) which mirrors the expression of the well-characterized angiogenic factor, *Vegf* (Figure 1c). On the other hand, *Cfb* was expressed at a significantly higher level in P17 OIR retina as compared to that in the retina of age-matched mice that had been kept under normoxic conditions throughout this period (Figure 1d). As expected, *Vegf* was also significantly induced in the angiogenic retina (Figure 1e). The mouse model of laser-induced choroidal neovascularization (CNV) has been widely used to study the pathogenesis of subretinal neovascularization associated with nAMD [33,35]. Laser injury causes the rupture of the Bruch’s membrane which results in a pronounced local inflammatory response at day 4 post-laser followed by CNV at the subretinal space at day 7 post-laser [36]. Our results demonstrate a significant induction of *Cfb* expression in the retina 7 days post laser treatment, correlating with the angiogenic phase of CNV lesion development (Figure 1f). Similarly, a significant induction of *Cfb* expression was observed in the retinal pigment epithelium (RPE)/choroid complex at 4- and 7-days post-laser, correlating with inflammatory and angiogenic phases of CNV lesion development, respectively (Figure 1g). Together, these data demonstrate a positive correlation between *Cfb* expression levels and ocular angiogenesis in both physiological and pathological conditions.

### 2.2. CFB Promotes Angiogenesis in In Vitro Models of Angiogenesis

**Figure 2:**
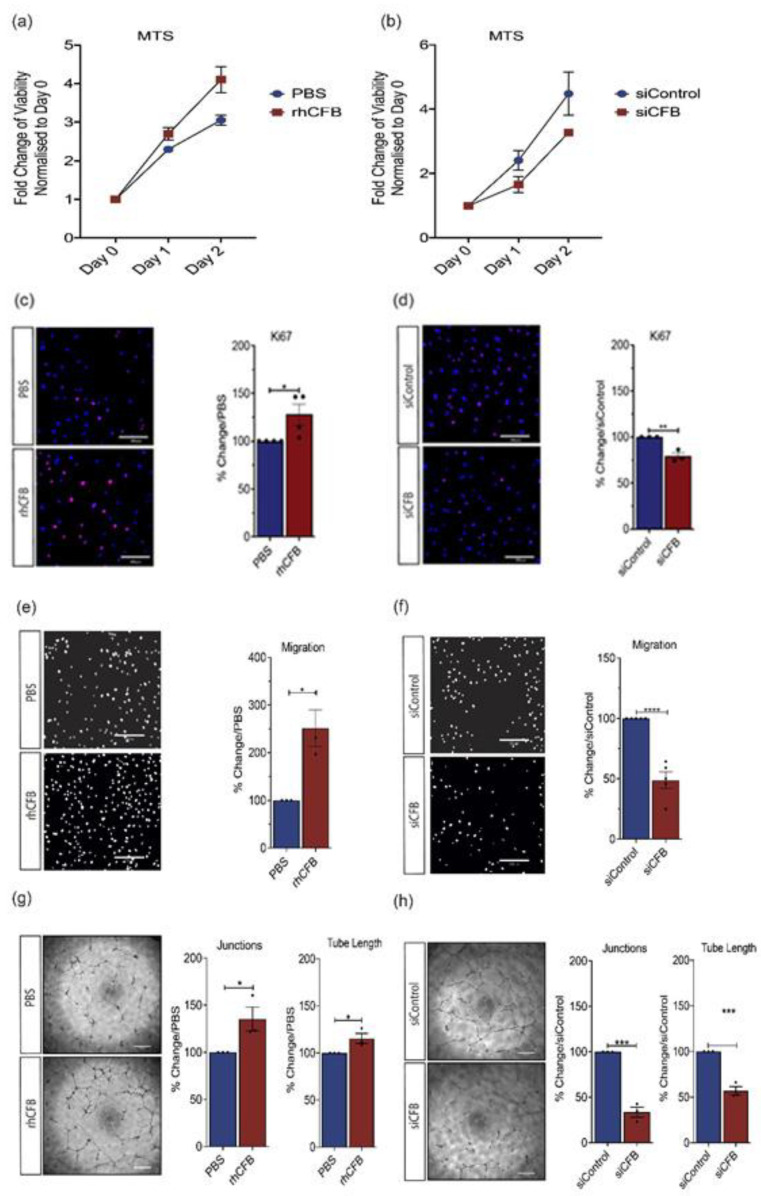
CFB promotes angiogenesis in in vitro models of angiogenesis. Growth curve of HRECS as measured by MTS following (**a**) treatment with 100 μg/mL rhCFB or vehicle control and (**b**) transfection with 100 nmol *siCFB* or 100 nmol siControl (n = 3 independent experiments). Representative images and quantitative analysis of Ki67^+^ (red) proliferating HRECs at 24-h following (**c**) 100 μg/mL rhCFB or PBS treatment and (**d**) transfection with 100 nmol *siCFB* or 100 nmol siControl (n = 3 independent experiments). Nucleus is stained with DAPI (Blue). Scale bar: 200 μM. Representative images and quantitative analysis of migrated HRECs as highlighted by DAPI staining (white) following (**e**) 24-h 100 μg/mL rhCFB or PBS treatment and (**f**) transfection with 100 nmol *siCFB* or 100 nmol siControl, (n = 3 independent experiments). Scale bar: 200 μM. Representative images and quantitative analysis of HREC tube formation in growth factor reduced Matrigel^®^ at 16-h following (**g**) 100 μg/mL rhCFB or PBS treatment and (**h**) transfection with 100 nmol *siCFB* or 100 nmol siControl (n = 3 independent experiments). Scale bar: 200 μM. Results represent the mean ± SEM. Statistical significance was determined by unpaired, two-tailed Student’s *t*-test; *: *p* < 0.05, **: *p* < 0.01, ***: *p* < 0.001, ****: *p* < 0.0001.

Having established an association between *Cfb* gene expression and ocular angiogenesis, we next evaluated the angiogenic potential of CFB using recombinant human CFB (rhCFB) or small interfering RNA-mediated CFB (*siCFB*) knockdown in human retinal ECs (HRECs). Compared with the vehicle-treated control, phosphate buffered saline (PBS), rhCFB significantly increased the number of viable cells as compared to Day 0 (Figure 2a), as determined by the MTS assay. On the other hand, *siCFB* significantly reduced the number of viable cells as compared with scrambled control siRNA (siControl) (Figure 2b). We next investigated the impact of rhCFB on cell proliferation using an antibody specific to, Ki-67, a nuclear protein that is associated with cell proliferation [37]. Consistent with the observation in HREC viability, rhCFB treatment resulted in a significant increase in the percentage of Ki-67^+^ cells compared to the vehicle control (Figure 2c), whereas *siCFB* significantly attenuated the percentage of Ki-67^+^ HREC compared to siControl (Figure 2d). Angiogenesis is a highly dynamic process: in response to local angiogenic cues, ECs degrade the basement membrane and migrate along chemical gradients established by angiogenic factors [38]. The Transwell migration assay was chosen to evaluate the effect of rhCFB on HREC migration with transmigrated HRECs labeled with 4′,6-diamidino-2-phenylindole (DAPI). Compared with the vehicle control, rhCFB significantly increased the number of HRECs that migrated across the Transwell (Figure 2e). On the contrary, compared with HRECs treated with siControl, *siCFB* transfected HRECs were less migratory (Figure 2f). The innate ability of ECs to spontaneously assemble into tubular structures is a characteristic feature of sprouting angiogenesis. Compared with the vehicle-treated controls, rhCFB was able to promote the ability of HREC to form a tube-like network as demonstrated by a significant increase in the number of junctions and total tube length (Figure 2g). Compared with those treated with siControl, the ability of HRECs subjected to siRNA-mediated *CFB* knockdown to form a tube-like structure was significantly attenuated (Figure 2h).

### 2.3. CFB Promotes Angiogenesis in Ex Vitro Models of Angiogenesis

**Figure 3:**
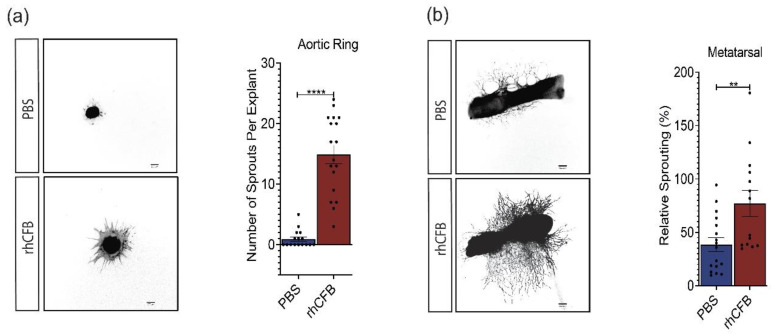
CFB promotes angiogenesis in ex vivo models of angiogenesis. (**a**) Representative images and quantitative analysis of vessel outgrowth from aortic ring explants labelled with I-B4 following 100 μg/mL rhCFB treatment (n = 18) or PBS treatment (n = 16). Scale bar: 200 μM. (**b**) Representative images and quantitative analysis of vessel outgrowth from metatarsal explants labelled with CD31 following 100 μg/mL rhCFB treatment (n = 16) or PBS treatment (n = 17). Scale bar: 200 μM. Results represent the mean ± SEM. Statistical significance was determined by unpaired, two-tailed Student’s *t*-test; **: *p* < 0.01, ****: *p* < 0.0001.

Angiogenesis is a complex process involving multiple cell types and extracellular components [39,40,41]. Ex vivo angiogenesis assays could reliably mimic blood vessel formation in vivo compared to in vitro single cell-based assays. In this study, rhCFB was first used to treat aortic explants isolated from neonatal mice. In comparison to the vehicle controls, rhCFB significantly promoted the number of sprouts from aortic rings as visualized by I-B4 Iseolectin staining (Figure 3a). Similarly, rhCFB led to a substantial increase in vessel outgrowth from the metatarsal explants as compared to the vehicle control, as demonstrated by the total vessel area labelled by CD31 (Figure 3b). Together, our data demonstrated a potent pro-angiogenic effect of CFB in ex vivo models of angiogenesis.

### 2.4. CFB Mediates VEGF Signaling in HRECs

**Figure 4:**
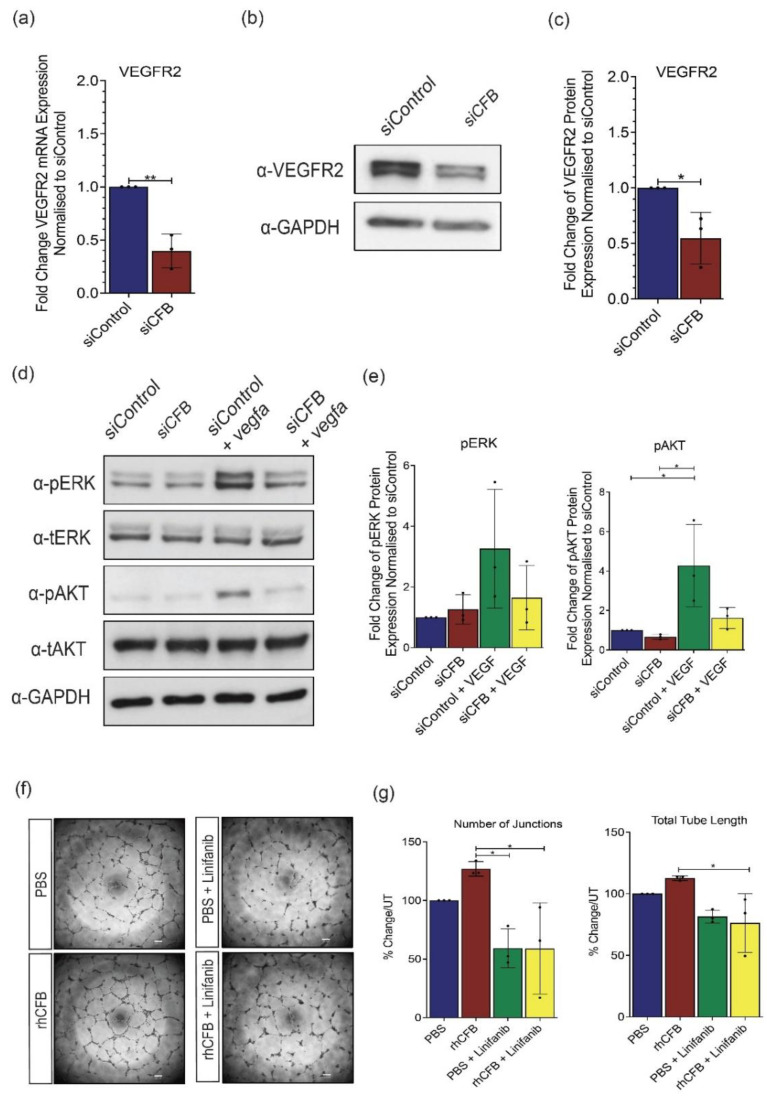
CFB promotes angiogenesis by mediating the VEGF signaling pathway. (**a**) Relative gene expression of VEGFR2 in primary HRECs transfected with 100 nmol *siCFB* or 100 nmol siControl, determined by RT-qPCR analysis (n = 3 independent experiments). (**b**) Representative Western blot and (**c**) densitometry analysis of VEGFR2 in primary HRECs transfected with 100 nmol *siCFB* or 100 nmol siControl (n = 3 independent experiments). Glyceraldehyde-2-phosphate dehydrogenase (GAPDH) was used as a loading control. (**d**) Representative Western blot and (**e**) densitometry analysis of pERK and pAKT in primary HRECs transfected with 100 nmol *siCFB* or 100 nmol siControl in the presence or absence of 50 ng/μL VEGFA (n = 3 independent experiments). GAPDH was used as a loading control. (**f**) Representative images and (**g**) quantification of HREC tube formation in Matrigel^®^ following 16-h treatment with 100 μg/mL rhCFB or vehicle control with the presence or absence of VEGFR2 inhibitor Linifanib (n = 3 independent experiments). Scale bar: 200 μM. Results represent the mean ± SEM. Statistical significance was determined by unpaired, two-tailed Student’s *t*-test or one-way ANOVA followed by Tukey multiple comparisons test; *: *p* < 0.05, **: *p* < 0.01.

Having established the pro-angiogenic role of CFB, we moved on to investigate its mechanism of action. VEGF serves as one of the most potent angiogenic stimulators [42] and therefore we investigated whether CFB exerts its function through mediating the VEGF-VEGF receptor 2 (VEGFR2) signaling cascade. Compared with siControl, a significant decrease in VEGFR2 gene expression (Figure 4a) and protein expression (Figure 4b,c) was observed in *siCFB*-treated HRECs. To further investigate this mechanistically, we also examined downstream signaling effectors of VEGFR2, phosphorylated ERK (p-ERK), and phosphorylated AKT (p-AKT). Our results showed that the promoting effect of VEGF on ERK and AKT phosphorylation is significantly compromised whereas the total ERK and AKT levels remain unchanged in *siCFB* treated HRECs (Figure 4d,e). To further understand the involvement of VEGFR2 in CFB-regulated angiogenesis, the ability of rhCFB to promote HREC tube formation in Matrigel, in the presence or absence of a VEGFR2 small molecule inhibitor Linifanib, was studied. Compared with the vehicle control, the promoting effect of rhCFB in HREC tube formation was significantly attenuated by Linifanib (Figure 4f,g). Together, our results showed that CFB promotes angiogenesis in a VEGF-dependent manner.

## 3. Discussion

Recent advances in understanding the molecular mechanisms of angiogenesis led to the identification of VEGF, one of the most potent stimulators of angiogenesis. Drugs that target VEGF have revolutionized the treatment of ocular angiogenic diseases. However, anti-VEGF drugs are not universally effective which is likely due to the action of VEGF-independent pathways following anti-VEGF treatment. Besides being a front-line surveillance system in host defense, the complement system, in particular the AP, is associated with vascular pathologies in the eye [32]. Specifically, polymorphisms in CFB have been associated with nAMD and retinopathy [25,26,43]. In addition, several groups have been trying to dissect the causative role of CFB in ocular angiogenesis using animal models [26,27,28,30]. However, the exact pathogenic significance of this association remains unclear. It is likely due to the complex interplay between immune response and angiogenesis in in vivo environment. The overarching aim of this study was to specifically explore the role of CFB in ocular angiogenesis using cell-based and organ culture models.

In this study, we first demonstrated the association between CFB and ocular angiogenesis in animal models of ocular angiogenesis. Hypoxia is a strong inducer of new blood vessel formation, whereas hyperoxia has been shown to arrest angiogenesis. Similar to VEGF, CFB mRNA expression is significantly suppressed in the retina of mice subjected to 5 days of hyperoxia treatment in a mouse model of OIR. On the other hand, in a similar manner to VEGF, retinal CFB expression is significantly induced at the angiogenic phase of OIR. Similarly, we observed a gradual induction of CFB in the developing mouse retina. These results suggest an association of CFB with retinal neo-vessel formation. Besides the neuroretina, abnormal blood vessels can also develop from the choroidal vessels underneath the neuroretina, which is the hallmark feature of nAMD. Using a mouse model of laser-induced CNV we observed an induction of CFB mRNA expression in both the retina and RPE/choroid complex at day 7 post-laser, a stage with active angiogenesis. These observations strongly support the link between CFB and choroidal neovascularization in the eye. It is worth noting that CFB expression is also highly induced in the RPE/choroid complex at 4 days following laser injury, a stage where there is a massive immune cell infiltration at the injury site. The massive inflammation induced in the CNV model and the potential role of CFB in ocular inflammation may explain the different responses of *Cfb^−/−^* mice to angiogenesis in CNV and OIR models as demonstrated in previous studies.

To investigate CFB’s role in ocular angiogenesis in a controlled environment, several in vitro and ex vivo angiogenesis assays were conducted. They revealed a critical role of CFB on HREC survival, proliferation, migration, and the ability to assemble into tubular networks. Besides endothelial cells, non-vascular cells and extracellular matrix components also contribute to new blood vessel formation. To study CFB’s role in angiogenesis in a multicellular environment, two ex vivo assays were carried out: the aortic ring and fetal metatarsal explant assays. Consistent with observations from the in vitro studies, vessel outgrowth from both aortic and metatarsal explanted tissue was significantly increased after the addition of exogenous CFB. These data provide strong support for the pro-angiogenic role of CFB.

The classic role of CFB is to contribute to the formation of C3/C5 convertase of the AP [19]. Here, our data revealed a positive correlation of CFB and VEGF expression levels in a mouse model of OIR. We further showed a reduced VEGFR2 mRNA and protein expression following CFB knockdown in HRECs which suggests that CFB mediates VEGFR2 expression at the transcriptional and possibly the translational level. As expected, CFB knockdown leads impaired downstream VEGF signaling in HRECs. Since a small molecule tyrosine kinase inhibitor for VEGFR2, Linifanib, is able to attenuate the promoting effect of rhCFB on HREC tube formation in Matrigel^®^, the proangiogenic function of CFB is, at least partially, dependent on the activation of VEGF signaling. However, it is not clear how CFB regulates VEGFR2 expression and VEGF signaling. Further investigation using RNA sequencing or proteomics studies should be carried out to delineate the regulatory control of VEGF by CFB. It will also be interesting to see whether CFB interacts directly with VEGF and its receptors by co-immunoprecipitation assay or surface plasmon resonance. The membrane attack complex (MAC), the end-product of complement activation, was previously reported to play essential roles in the development of laser-induced CNV [29], possibly through regulating the expression of angiogenic factors, including VEGF [44]. Whether CFB-mediated VEGF expression, the activation of the downstream signaling pathway, and angiogenic response are MAC-dependent warrants further investigations. CFB is a secreted protein that is mainly generated by hepatic cells of the liver; however, it has also been reported to be expressed in other types of cells, including retina pigmented epithelial (RPE) cells [45]. Here, we showed that CFB is generated by retinal ECs, and siRNA-mediated knockdown of CFB leads to compromised EC functions. Since multiple types of ocular cells contribute to CFB production, it is possible that CFB acts in both paracrine and autocrine manner on retinal ECs.

**Figure 5:**
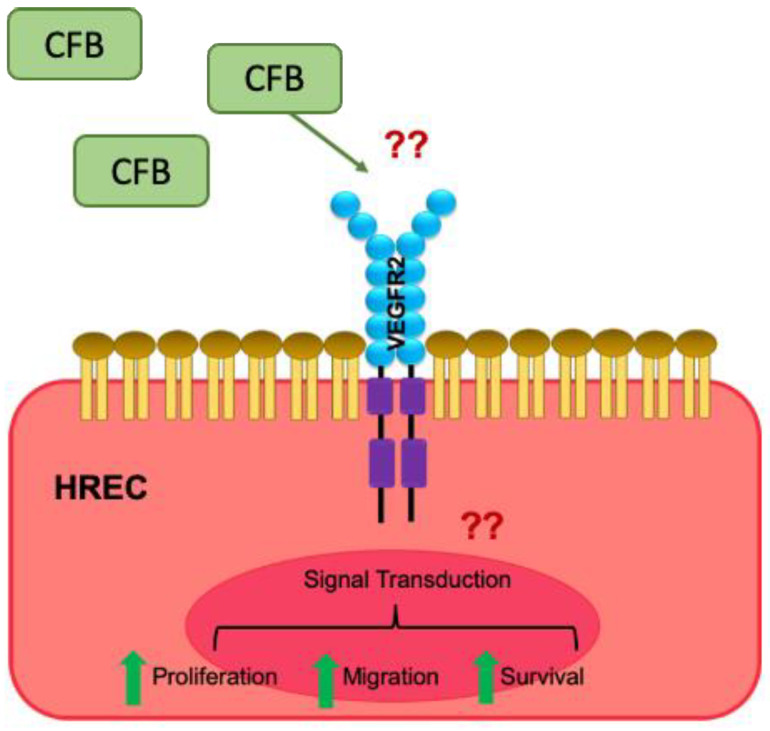
Schematic representation of the proposed mechanism of action of CFB. The inflammatory microenvironment in DR increases the availability of CFB, which acts in a paracrine manner on retinal ECs, inducing the VEGF signaling cascade, resulting in enhanced EC survival, proliferation, migration, and network formation. ‘??’ indicate the unknown factors that mediate the effect of CFB.

In summary, our work presented evidence on the association of CFB and ocular angiogenesis in different preclinical models. We established the causative role of CFB in angiogenesis in HREC-based in vitro assays and in ex vivo models of angiogenesis. This novel function is distinct from CFB’s well-established role within the alternative pathway and suggests that CFB might not only contribute to the innate defense mechanism of the alternative pathway but also have a direct impact on ocular neovascularization. Considering the upregulation of CFB during developmental angiogenesis of the mouse retina, we could not rule out the involvement of CFB in physiological neovascularization. Finally, we showed that CFB exerts its role in angiogenesis in a VEGF-dependent manner (Figure 5). Together with its role in inflammation, there is a need to carefully assess CFB as a druggable target. Extra attention should be paid to possible side effects of targeting CFB for physiological processes that require angiogenesis such as wound healing.

## 4. Materials and Methods

### 4.1. Animals

C57BL/6 mice were purchased from the InVivos Pte Ltd., (Singapore). All animal procedures were reviewed and approved Singapore and Institutional Animal Care and Use Committee of Agency for Science (IACUC), Technology and Research (A*STAR) (IACUC, Protocol number: 181334).

### 4.2. Cell Culture

Primary HRECs (Angio-Proteomie, Boston, MA, USA) were cultured in endothelial growth medium-2^TM^ (EGM-2^TM^) (Angio-Proteomie, USA) containing 10% fetal bovine serum (FBS), recombinant growth factors, and 1× penicillin and streptomycin (P/S). Cells were grown on Quick Coat Solution (Angio-Proteomie, USA) coated plates and maintained in humidified 5% CO_2_/95% air at 37 °C. Freestyle Human Embryonic Kidney 293 cells (HEK293F) (Life Technologies, Carlsbad, CA, USA) were maintained in Freestyle 293F Expression Medium (Life Technologies, Carlsbad, CA, USA)on an orbital shaker (130 rpm) in humidified 5% CO_2_/95% air at 37 °C.

### 4.3. RNA Extraction and Quantitative Real-Time PCR

Total RNA was extracted from homogenized mouse tissues or homogenized cell lysates using NucleoSpin RNA, Mini Kit (Macherey-Nagel, Düren, Germany) following the manufacturer’s instructions. cDNA was synthesized using qscript cDNA Supermix (Quanta BioSciences, Beverly, MA, USA) according to the manufacturer’s instructions. Quantitative real-time PCR was performed in a total volume of 20 μL containing.

PrecisionFast 2× qPCR Mastermix (with SYBR green and low ROX) (Primerdesign, Camberley, UK) using a QuantStudio^TM^ 6 Flex Real-Time PCR System (Thermo Fisher Scientific, Carlsbad, CA, USA). Primer sequences used in this study are listed in (Table 1). The expression levels of respective target genes were normalized to β-actin, and relative gene expression was calculated using the standard 2^−^^ΔΔCT^ method.

### 4.4. Protein Extraction and Western Blot Analysis

Cells were lysed on ice in RIPA buffer containing 1 mM dithiothreitol (DTT) (Sigma-Aldrich, St Louis, MO, USA), 1× protease inhibitor (Roche, Basel, Switzerland), 1× phosphatase inhibitor (Sigma-Aldrich, USA), and 1 mM phenylmethylsulfonyl fluoride (PMSF) (Sigma-Aldrich, St. Louis, MO, USA) before being centrifuged at 13,000 rpm for 10 min at 4 °C. The supernatants were subjected to Bio-Rad Protein Assay (Bio-Rad, Hercules, CA, USA) for total protein analysis before being separated via sodium dodecyl sulfate-polyacrylamide gel electrophoresis (SDS-PAGE) and transferred onto an Immobilon-PSQ 0.2 μm Polyvinylidene Fluoride (PVDF) Membrane (Merck Millipore, Burlington, MA, USA). Blots were probed with VEGFR2 antibody (rabbit polyclonal, ab1661, Abcam, UK, RRID:AB_883437), p-ERK antibody (rabbit monoclonal, 4376, CST, USA, RRID: AB_331772), t-ERK antibody (rabbit polyclonal, 9122, CST, USA, RRID: AB_ 823567), p-AKT antibody (rabbit monoclonal, 9271, CST, USA, RRID: AB_329825), t-AKT antibody(rabbit polyclonal, 9272, CST, USA, RRID:AB_329827) and GAPDH antibody (mouse monoclonal, sc-3223, Santa Cruz Biotechnology, Inc., Dallas TX, USA, RRID:AB_627679), followed by horseradish peroxidase (HRP)-conjugated secondary antibodies (Bethyl Laboratories, Inc., Montgomery, TX, USA).

### 4.5. Molecular Biological Methods

The coding sequence of human CFB (NM_001710.5) carrying a 6XHis tag at the 3′ end and Kozak consensus sequence at the 5′ end, was cloned into pcDNA3.1 at the AFLII/XbaL sites to form pcDNA-CFB-His construct. rhCFB protein was expressed in Freestyle 293F cells (Life Technologies, UK). Conditioned media from Freestyle 293F cells transfected with the CFB-His construct was collected and concentrated using an Amicon^®^ Pro Affinity concentrator (Merck, Millipore, Burlington, MA, USA) before being equilibrated with binding buffer. Ni Sepharose high performance nickel-charged immobilized metal ion affinity chromatography (IMAC) resin (Merck, Millipore, Burlington, MA, USA) was used to purify CFB-His recombinant protein which was buffer exchanged with ice cold 1x PBS using a Scientific Slide-A-Lyzer MINI Dialysis Device (Thermo Fisher Scientific, USA) and further concentrated using an Amicon^®^ Ultra 2 mL centrifugal filter (Merck, Millipore, Burlington, MA, USA). Protein purity was confirmed by Bio-Safe™ Coomassie Stain (Bio-Rad, Hercules, CA, USA).

siRNA oligonucleotides (cat. no. L-005792-00-0010; Dharmacon) were used for human CFB gene knockdown, while control siRNA (cat. no. D-001810-10-20; Dharmacon) was used as a negative control. Transfection was performed using Lipofectamine 3000 (Invitrogen) according to the manufacturer’s protocol.

### 4.6. Mouse Model of Laser-Induced Choroidal Neovascularisation

CNV was induced in 6–8-week old mice as described [46], and the eyes were harvested from mice at 0 (before laser treatment), 4, 7, 14, 21, and 35 days post laser. After enucleation retinae were immediately dissected and snap-frozen for gene expression analysis.

### 4.7. Mouse Model of Oxygen-Induced Retinopathy (OIR)

OIR was induced as described. In brief, postnatal day (P) 7 mice and nursing mothers were placed in a 75% oxygen chamber for 5 days and exposed to a standard 12 h light-dark cycle. Mice were returned to room air at P12. Eyes were harvested from mice at P11 (immediately after removal of mice from the hyperoxic chamber) and at P17. After enucleation, retinae were immediately dissected and snap-frozen for gene expression analysis.

### 4.8. Aortic Ring Assay

The aortic ring assay was carried out using a modified method as described previously [47]. Briefly, thoracic aortas were dissected from P3 mice and cut into rings of approximately 1 mm in diameter. A single aortic ring was embedded per 96-well coated with rat tail collagen gel (BD Biosciences, San Jose, CA, USA). Explants were incubated for 30 min at 37 °C to allow for complete polymerization of the collagen gel. After incubation, aortic ring explants were cultured in 100 uL of OptiMEM supplemented with 2% FBS and 1× P/S for 24 h. The following day, media was replaced with OptiMEM supplemented with 2% FBS and 1× penicillin and streptomycin containing 100 μg/mL rhCFB or vehicle control. Media was replaced every 2 days. On day 10 of culture, the explants were fixed and stained with Griffonia Simplicifolia isolectin B4 (IB4) (Vector Lab, Burlingame, CA, USA). Vessel outgrowth was visualized using Eclipse Ti-E Inverted Research Microscope (Nikon, Tokyo, Japan). The IB4^+^ sprouts were counted manually in real-time. The focus was adjusted manually during counting, to ensure vessels sprouting in different planes were counted.

### 4.9. Metatarsal Sprouting Assay

The fetal metatarsal sprouting assay was carried out as described previously [47]. Briefly, embryos between E16.5 to E18 were sacrificed and metatarsal bones were dissected. Individual metatarsals were mounted onto a 24-well coated with 0.1% gelatin and incubated for 10 min to allow explants to adhere. Explants were cultured in 200 μL DMEM supplemented with 10% FBS and 1× P/S for 24 h. The following day, media was replaced with DMEM supplemented with 10% FBS and 1× P/S containing 100 μg/mL rhCFB or vehicle control. Media was replaced every 2 days. On day 10 of culture, the explants were fixed and stained with primary antibody against CD31 (rat monoclonal antibody, 553370, BD Biosciences, San Jose, CA, USA, RRID:AB_394816), followed by incubation with secondary antibody Alexa Fluo 488 (A-11006, Thermo Fisher Scientific, USA).

### 4.10. Viability Assay

HRECs were seeded at a density of 1 × 10^3^ cells per 96-well in 100 μL EGM-2^TM^, and cultured for 24 h. Cells were washed once with 1× PBS and treated with EGM-2^TM^ media containing 100 μg/mL rhCFB or vehicle control for 48 h. Viability was evaluated using the colorimetric MTS tetrazolium assay. At 0 (immediately after adding treatment), 24 h, and 48 h, 10 μL of MTS reagent was added to each 96-well and incubated for 3 h. After incubation, the absorbance of the formazan product was measured using a plate reader at the wavelength of 490 nm. The final value was obtained by subtracting the absorbance reading of a blank media control.

### 4.11. Proliferation Assay

HRECs were seeded at a density of 4 × 104 cells per 48-well in 300 μL EGM-2^TM^, and cultured for 24 h. Cells were washed once with 1× PBS and treated with EGM-2^TM^ media containing 100 μg/mL rhCFB or vehicle control for 48 h. Cells were then fixed and stained with primary antibody against Ki67 (rabbit monoclonal antibody, ab-16667, Abcam, UK, RRID: AB_302459) overnight at 4 °C for detection of proliferating cells, followed by incubation with secondary antibody Alexa Fluo 488 (A-11008, Theremo Fisher Scientific, USA) and DAPI (Thermo Fisher Scientific, USA) for visualization of cell nuclei. Fluorescent images were captured using Eclipse Ti-E Inverted Research Microscope (Nikon, Japan). Images were captured across five fields of views per well. The cell proliferation rate was evaluated as the percentage of Ki67-positive cells. The average of five fields of view was taken to give a final proliferation rate per well.

### 4.12. Matrigel Tube Formation Assay

Growth factor-reduced Matrigel^®^ is a specially formulated ECM that supports cells to form two-dimensional vascular networks. Matrigel^®^ Growth Factor Reduced Basement Membrane Matrix (Corning) was added to the wells of a 96-well plate and incubated for 30 min at 37 °C for polymerization. HRECs were seeded onto polymerized Matrigel^®^ at a density of 1.2 × 103 cells per 96-well plate in 100 μL EGM-2^TM^ containing either 100 μg/mL rhCFB or vehicle control, and cultured overnight for 16 h. After incubation cells were imaged under phase contrast with Eclipse Ti-E Inverted Research Microscope (Nikon, Japan) at 4x magnification. Quantification of the number of junctions and total vessel length was carried out using the Image J (version) (National Institute of Health, Bethesda, MD, USA) Angiogenesis Analyzer (Gilles Carpentier).

### 4.13. Transwell Migration Assay

Transwell inserts (8 μM) were coated with 100 μg/mL rat tail collagen (BD Biosciences, USA) and incubated overnight before washing once with 1× PBS and air drying for 1 h. HRECs were seeded at a density of 3 × 104 cells per upper chamber of transwell inserts in 200 μL endothelial basal media-2^TM^ (EBM-2^TM^) containing either 100 μg/mL rhCFB or vehicle control. EBM-2^TM^ containing 5% FBS was used as a chemoattractant in the lower chamber of the transwell inserts. After 24 h incubation, migrated cells were fixed in 1% PFA and stained with DAPI (Thermo Fisher Scientific, USA) for visualization of cell nuclei. Images were captured across five fields of view per well with Eclipse Ti-E Inverted Research Microscope (Nikon, Japan). The total number of cell nuclei per field of view was counted manually using Image J (National Institutes of Health, USA). The average of five fields of view was taken to give a final number of migrated cells per well.

### 4.14. Statistical Analysis

All data are shown as mean ± SEM. Three independent repeats were carried out for each experiment unless stated otherwise. One-way ANOVA followed by Turkey’s post hoc analysis or unpaired two-tailed Student’s *t*-test was used to determine the statistical significance using GraphPad Prism 6 (GraphPad Software, San Diego, CA, USA). * *p* < 0.05; ** *p* < 0.01; *** *p* < 0.001; **** *p* < 0.0001.

## 5. Conclusions

Our study established a positive association of CFB and ocular angiogenesis, demonstrated the causative role of CFB in angiogenesis, and showed that CFB exerts its function in a VEGF-dependent manner.

## Figures and Tables

**Table 1:** Sequences of the forward and reverse primers used for gene expression analysis.

Target (Mouse)	Forward Sequence (5′-3′)	Reverse Sequence (5′-3′)
*β-actin*	CTGACGGCCAGGTCATCACT	TAGTTTCATGGATGCCACAGGAT
*Vegf-a*	TAGAGTACATCTTCAAGCCG	TCTTTCTTTGGTCTGCATTC
*Cfb*	GCATGGTGTGGGAGCATAAA	GGCTTGCCATGGTTGCTTA
**Target (Human)**	**Forward Sequence (5′-3′)**	**Reverse Sequence (5′-3′)**
RPLPO	CCTTCTCCTTTGGGCTGGTCATCCA	CAGACACTGGCAACATTGCGGACAC
CFB	GGAAGGGAATGTGACCAGG	AAGGCAGGAGAGAAGCTGG
VEGFR2	CCAGCAAAAGCAGGGAGTCTGT	TGTCTGTGTCATCGGAGTGATATCC

## Abbreviations

CFB	Complement Factor B
AP	Alternative Pathway
CNV	Choroidal Neovascularization
OIR	Oxygen Induced Retinopathy
EC	Endothelial Cell
ECM	Extracellular Matrix
DR	Diabetic Retinopathy
nAMD	Neovascular Age-Related Macular Degeneration
ROP	Retinopathy of Prematurity
VEGF	Vascular Endothelial Growth Factor
CFD	Complement Factor D
CFH	Complement Factor H
P	Postnatal Day
SEM	Standard Error of Mean
rhCFB	Recombinant CFB
siCFB	Small Interfering RNA-Mediated CFB Knockdown
HREC	Human Retinal Endothelial Cell
PBS	Phosphate Buffered Saline
siControl	Small Interfering RNA-mediated Scrambled Control
DAPI	4′,6-Diamidino-2-Phenylindole
VEGFR2	Vascular Endothelial Growth Factor Receptor-2
p-ERK	Phosphorylated ERK
p-AKT	Phosphorylated AKT
MAC	Membrane Attack Complex
RPE	Retinal Pigment Epithelium
IACUC	Institutional Animal Care and Use Committee
A*STAR	Agency for Science Technology and Research
EGM-2^TM^	Endothelial Growth Media-2^TM^
FBS	Fetal Bovine Serum
P/S	Penicillin Streptomycin
HEK293F	Freestyle Human Embryonic Kidney 293 Cells
DTT	Dithiothreitol
PMSF	Phenylmethylsulfonyl Fluoride
SDS-PAGE	Sodium Dodecyl Sulphate–Polyacrylamide Gel Electrophoresis
PVDF	Polyvinylidene Fluoride
HRP	Horseradish Peroxidase
IMAC	Immobilized Metal ion Affinity Chromatography
IB4	Isolectin B4
EBM-2^TM^	Endothelial Basal Media-2^TM^
